# Cervical cancer in Ethiopia – predictors of advanced stage and prolonged time to diagnosis

**DOI:** 10.1186/s13027-019-0255-4

**Published:** 2019-11-11

**Authors:** Matthias Begoihn, Assefa Mathewos, Abreha Aynalem, Tigeneh Wondemagegnehu, Ulrike Moelle, Muluken Gizaw, Andreas Wienke, Christoph Thomssen, Dawit Worku, Adamu Addissie, Ahmedin Jemal, Eva Johanna Kantelhardt

**Affiliations:** 1 Department of Gynecology, Martin-Luther-University, Halle (Saale), Germany; 2 Radiotherapy Center, School of Medicine, Addis Ababa University, Addis Ababa, Ethiopia; 3 Institute of Medical Epidemiology, Biostatistics and Informatics, Martin-Luther-University, Halle (Saale), Germany; 4 Department of Preventive Medicine School of Public Health, Addis Ababa University, Addis Ababa, Ethiopia; 5 Department of Gynecology, School of Medicine Addis Ababa University, Addis Ababa, Ethiopia; 6 Department of Intramural Research, American Cancer Society, Atlanta, Georgia

**Keywords:** Cervical cancer, Sub-Saharan Africa, Patient interval, Ethiopia, HIV

## Abstract

**Introduction:**

In Ethiopia, most cervical cancer patients present at advanced cancer stages, long time after they experience first symptoms. We investigated possible predictors of long time spans between symptom onset and pathologic diagnosis (patient intervals). We also aimed to seek out predictors for advanced cancer stage diagnosis.

**Methods:**

We conducted a retrospective cohort study among 1575 cervical cancer patients who were registered at Tikur Anbessa Specialized Hospital (TASH), Addis Ababa, Ethiopia between September 2008 and September 2012. Cox proportional hazards regression was used to find predictors of long patient intervals. Cumulative odds ordinal logistic regression was used to identify predictors of cancer stage at diagnosis.

**Results:**

Median patient interval was 30 weeks, with the interval substantially longer in patients residing in rural than urban areas. Longer patient intervals were associated with more advanced cancer stages at pathologic diagnosis. HIV-positive women had an almost 1.5 times increased risk of diagnosis at a more advanced stage.

**Conclusion:**

Cervical cancer patients are diagnosed after long time periods leading to advanced stages at diagnosis. Measures to raise awareness about cervical cancer, to increase screening and to shorten the time interval from recognition of symptoms to diagnosis are urgently needed.

## Introduction

Cervical cancer incidence and mortality has been drastically reduced in high resource countries during the last decades. This can be largely attributed to the implementation of screening programs for the detection of precancerous lesions and HPV and improved therapy [1, 2]. Yet in low- and middle income countries where access to such measures is limited, cervical cancer remains a significant health problem. The vast majority of an estimated number of 311.000 cervical cancer deaths worldwide occur in less developed regions [3]. In Ethiopia, where almost 6.300 new cases are diagnosed annually, about 4.884 women die from cervical cancer each year. This makes cervical cancer the second-most common cancer in the country, and the second-most deadly cancer among Ethiopian women [4].

One of the most important prognostic factors is stage at diagnosis, linking early-stage diagnosis with better chances of survival [5]; still most cervical cancer patients present at advanced stages in Ethiopia [6]. Studies examining predictors of late and advanced stage presentation of cervical cancer patients in low- and middle-income countries have been scarce [7]. The relationship between HIV-infection and cervical cancer and the question of whether HIV-infection leads to more advanced cancer stages is discussed controversially [8–10]. The timespan between symptom onset and diagnosis has been associated with stage at diagnosis [11], but other studies could not confirm this [12, 13]. However, these studies were conducted in high-income countries where time to diagnosis is considerably shorter. It is unclear whether these results likewise apply to low-income countries such as Ethiopia, where time to diagnosis is long and patients present at advanced stages. Tragically, in a previous study of a hospital cohort of 1.059 cervical cancer patients receiving oncologic treatment in Addis Ababa, Ethiopia, we found long periods of time between diagnosis and the beginning of cancer treatment. This led to stage-migration and thus decreased chances of survival [6].

In this study, using the same cohort (with the addition of patients who were diagnosed with cervical cancer but never received therapy) we focused on the time between patient reported onset of symptoms and pathological diagnosis (patient interval). The aim of this study was to find predictors for cancer stage at pathological diagnosis and longer patient intervals in Ethiopia. We further hypothesized that longer patient intervals lead to more advanced stages at diagnosis.

## Methods

### Setting

We conducted a retrospective cohort study among cervical cancer patients who registered at Tikur Anbessa Specialized Hospital (TASH), Addis Ababa, Ethiopia between September 2008 and September 2012 as described earlier [6, 14, 15]. TASH is the largest hospital in Ethiopia and the only hospital in the country currently offering radiotherapy – thus, people from all parts of the country were referred there for therapy. Early tumor stages from FIGO Ia - IIa were treated with radical hysterectomy with curative intentions. More advanced tumor stages and cases of unclear surgical margins were treated with external beam radiotherapy. At the time of the study, brachytherapy as recommended according to international guidelines was not available in Ethiopia.

### Study population

Ethiopian woman who presented at TASH between September 2008 until September 2012 with a primary diagnosis of invasive cervical cancer were eligible for this study. Of 1655 collected patient files, we used 1575 cases for further analysis. Of these 80 patients were excluded: 42 patients presenting with recurrent disease; two asymptomatic patients who were incidentally diagnosed with cervical cancer; 35 with missing dates regarding pathological diagnosis or symptom onset; one with noninvasive cancer. Since there was no nationwide cervical cancer screening program in place, all women included presented with symptomatic disease. Information regarding patient characteristics, clinical characteristics such as histology, FIGO-Stage, symptoms and waiting times were retrieved from patient files from the oncology and gynecology ward.

### Predictor variables

The patients’ residency was classified as urban or rural. Patients living in one of the 10 largest cities in Ethiopia were classified as ‘urban’, while the remaining patients living in smaller cities and villages were classified as ‘rural’. HIV-status was subdivided into two groups: positive HIV-status, and negative or unknown HIV-status. Comprehensive HIV-screening at TASH was routinely introduced after September 2011; before this time, only clinically-suspicious patients or patients with a high risk profile (e.g. those with an HIV-positive partner) were screened for HIV. The predictor variables in both models were preselected using variables that were coherent with similar studies [16–18]. Other risk factors commonly examined in regards to both late and advanced stage presentation include socioeconomic status variables such as low education and illiteracy [16–22]. These were not recorded in the patient files at TASH, and thus could not be assessed in this analysis.

### Staging

Tumors were staged according to guidelines set by the International Federation of Gynecology and Obstetrics (FIGO) [23]. Stage at primary diagnosis when first seen by a physician was used for further analysis in this study. FIGO-stages were assessed around the date of pathology report. In most cases, a chest X-Ray and abdominal ultrasound followed. If there was an upstaging within 4 weeks after the first staging due to distant metastasis findings or hydronephrosis, the higher FIGO-stage was used. FIGO-stages were later grouped for statistical analysis into stage of FIGO I - IIa (patients receiving primary surgery), FIGO IIb, FIGO III (FIGO IIIa and IIIb) and stage of FIGO IV (IVa and IVb).

### Time intervals

Patient interval was defined as the time interval between the date the patient noticed the first symptom and the date of the biopsy report. This interval was used because the date of the first symptom and the date of pathologic diagnosis were widely available, whereas the date of first presentation – i.e. when the patient was first seen by a clinician – was not documented for most patients. Data on symptom onset were abstracted from handwritten documents in the patient files. Patient interval was used as a continuous variable in weeks to avoid loss of power and bias [24, 25].

### Statistical analysis

Data were analyzed using SPSS Version 23. A cumulative odds ordinal logistic regression with proportional odds was conducted to examine the effect of time to diagnosis, HIV-status, place of residence and age on stage at diagnosis. The proportional odds assumption was assessed by a full likelihood ratio test. Odds ratios are presented with their corresponding 95% confidence intervals.

Cox proportional hazards regression was used to evaluate the association between predictors and the patient interval and to calculate hazard ratios (HRs) with 95% confidence intervals. Simple regression analysis was conducted using the predictor variables age, place of residence and HIV-status. For multiple regression analysis, we included all three variables into the model.

## Results

### Patient characteristics

Mean age was 49 years (SD ±11.6 years). Known HIV-seropositive women presented at a mean age of 39 years, while patients with a negative or unknown HIV-status presented at a mean age of 50 years. One hundred thirty-five of the patients were tested HIV-seropositive (8.6%). Out of the 494 women screened for HIV, 135 women were screened positive and 359 were screened negative. The rest of the women where not screened. Of the HIV-seropositive women, 86.3% were on antiretroviral medication. Close to two thirds of the women came from rural areas. Most woman presented with advanced stages (55.2% stage IIIb or higher). Only 12.1% presented with an early FIGO-stage of I-IIa, making them eligible for surgery (Table 1).

**Table 1:** Demographic and clinical characteristics of the study population according to FIGO Stage at diagnosis (*n* = 1575)

Patient Characteristics	FIGO Stage				
	All Stages	I-IIa	IIb	III	IV
	N	N (%)	N (%)	N (%)	N (%)
All Patients	1575	191 (12.1)	497 (31.6)	731 (46.4)	156 (9.9)
Age (years) (mean + SD) Range 21–93	48,9 ± 11,5	47.9 ± 11.4	49.6 ± 11.9	48.4 ± 11.3	50.9 ± 11.5
Menopausal status					
Premenopausal	344	41 (11.9)	102 (29.7)	175 (50.9)	26 (7.6)
Postmenopausal	1212	148 (12.2)	386 (31.8)	548 (45.2)	130 (10.7)
Unknown	19	2 (10.5)	9 (47.4)	8 (42.1)	0 (0)
Residence					
Rural	976	114 (11.7)	303 (31.0)	465 (47.6)	94 (9.6)
Urban (Biggest 10 Cities)	599	77 (12.9)	194 (32.4)	266 (44.2)	62 (10.4)
HIV Status					
HIV-positive	135	11 (8.1)	37 (27.4)	74 (54.8)	13 (9.6)
negative / unknown	1440	180 (12.5)	460 (31.9)	657 (45.6)	143 (9.9)
Parity (mean + SD) Range 0–17	6.1 ± 3.0	5.6 ± 2.9	6.6 ± 3.1	6.0 ± 3.0	6.5 ± 3.0
Marital Status					
Unmarried	12	2 (6.7)	4 (33.3)	5 (41.7)	1 (8.3)
Early marriage (< 18 years)	1187	146 (12.3)	380 (32.0)	550 (46.3)	111 (9.4)
> 18 years / unknown age	94	20 (21.3)	30 (31.9)	38 (40.4)	6 (6.4)
Unknown Martial Status	282	23 (8.2)	83 (29.4)	138 (48.9)	38 (13.5)
Histology					
Squamous cell carcinoma	1488	170 (11.4)	462 (31.1)	711 (47.8)	145 (9.8)
Adenocarcinoma	66	17 (25.8)	29 (43.9)	12 (18.2)	8 (12.1)
Other	21	4 (19.0)	6 (28.6)	8 (38.1)	3 (14.3)

*FIGO* International Federation of Gynecology and Obstetrics, *SD* Standard deviation, *HIV* Human immunodeficiency virus

% as proportion among stages

### Predictors for longer patient interval

Median patient interval was 30 weeks (range 0–526 weeks). It was shorter for HIV-positive women (25 weeks) compared to women with a negative or unknown HIV-status (30 weeks). Rural women received their pathologic diagnosis after a median time of 32 weeks whereas women from one of the 10 largest cities in Ethiopia were diagnosed after a median time interval of 25 weeks.

Univariate analysis indicated a higher risk for longer patient intervals for women from rural areas compared with woman coming from one of the 10 largest cities (HR 1.23; CI 1.11–1.36) (Table 2). Also more likely to be diagnosed later in univariate analysis were younger patients (HR 0.99) and women with a negative or unknown HIV-status (HR 1.19, (CI 1.004–1.43)). After entering all three variables (age, place of residence, HIV-status) in the multiple Cox-Model, the adjusted Hazard Ratio for HIV-status was 1.1 (CI 0.91–1.32) and for age 0.99 (CI 0.99–1). The adjusted Hazard Ratio for place of residence remained 1.23 (CI 1.11–1.36).

**Table 2:** Predictors for longer patient interval (time between symptom onset and pathologic diagnosis)

Predictor	Unadjusted HR (95% CI)	Adjusted HR (95% CI)	*p*-value
Age (years)	0.99 (0.99–1)	0.99 (0.99–1)	0.7
Place of residence			
Rural	1.23 (1.11–1.36)	1.23 (1.11–1.36)	< 0.001
Urban			
HIV-Status			
positive	1.19 (1.004–1.43)	1.1 (0.91–1.32)	0.29
Negative/unknown	1	1	

*HR* Hazard ratio, *CI* Confidence interval, *HIV* Human immunodeficiency virus

### Predictors for more advanced stage at diagnosis

**Fig. 1:**
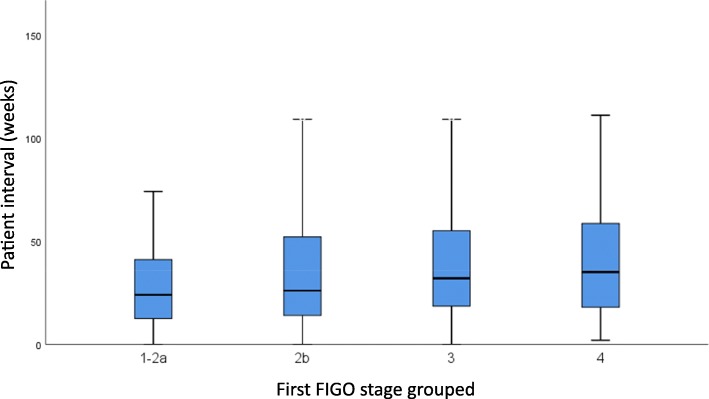
Box-plot showing median patient interval (time between symptom onset and pathologic diagnosis in weeks) with 25th and 75th percentile by FIGO stage group

We found longer patient intervals associated with more advanced FIGO-stages at diagnosis in the proportional odds model (OR 1.004 (CI 1.002–1.006) p: < 0.001) (Table 3). This means that the odds of being diagnosed in a more advanced stage group increased by 0,004 every week. Patient interval was shortest for early stages (24 weeks for FIGO I-IIa) and longest for advanced stages (35 weeks for FIGO IV) (Fig. 1).

**Table 3:** Predictors for more advanced stage at diagnosis

Predictors	Odds Ratio (95% CI)	*p*-value
Waiting time (weeks)	1.004 (1.002–1.006)	< 0.001
Age (years)	1.004 (0.99–1.01)	0.31
Place of residence		
rural	1.08 (0.89–1.31)	0.42
urban	1	
HIV-Status		
positive	1.48 (1.05–2.1)	0.025
Negative / unknown		

*CI* Confidence interval, *HIV* Human immunodeficiency virus

Known HIV-infection was associated with an almost 1.5-fold risk of diagnosis at a more advanced stage compared to those patients with a negative or unknown HIV-status (95% CI 1.05–2.1 *p* = 0.025). Our data suggested no association between place of residence, age and stage at diagnosis.

### Symptoms at diagnosis

All patients presented with symptoms related to cervical cancer, with the most common symptoms being abnormal vaginal bleeding, abdominal pain and vaginal discharge (Table 4). Even in early stages I-IIa, 91.1% of patients presented with abnormal vaginal bleeding. Constipation was suggestive of late stage disease: 64% of the patients presenting with constipation were staged IIIb and higher.

**Table 4:** Intensity of common symptoms when first seen by a physician among cervical cancer patients (*n* = 1575)

Symptoms	Intensity of symptoms									
	None		Mild		Moderate		Severe		Unknown	
	N	(%)	N	(%)	N	(%)	N	(%)	N	(%)
Vaginal bleeding	115	(7.3)	404	(25.7)	977	(62.0)	74	(4.7)	5	(0.3)
Pelvic pain	442	(28.1)	213	(13.5)	858	(54.5)	57	(3.6)	5	(0.3)
Vaginal discharge	424	(26.9)	331	(21.0)	814	(51.7)	–		6	(0.4)
Constipation	1267	(80.4)	101	(6.4)	197	(12.5)	5	(0.3)	5	(0.3)

## Discussion

We found that longer patient intervals increased the risk of advanced stage cervical cancer diagnosis. Previous studies on the effect of longer patient intervals on outcomes like advanced stage and impaired survival presented conflicting results. Consistent with our findings, in the 1980s Fruchter et al. reported an increased risk for presentation at advanced stages of cervical cancer after long patient intervals [11]. In contrast, Tokuda et al. in Japan found no association between patient interval and stage of cervical cancer in the 1990s. However, Japan is a high-resource country and median patient interval was only 30 days [12], differing substantially from the long patient intervals observed in Ethiopia.

We also found that HIV-infection was associated with more advanced cancer stages at time of diagnosis compared to patients with a negative or unknown HIV-status. The association of HIV and HPV is well-known, and previous studies repeatedly linked HIV-infection with a higher prevalence, incidence and persistence of HPV-infection and its progression into precancerous lesions (especially for patients with low CD4 cell counts) [26, 27]. However, the association between HIV and invasive cervical cancer is less clear. Published data indicate a 1.6 to 2.4 increased risk of developing invasive cervical cancer for HIV-positive women [28, 29]. The effect of seroprevalence of HIV on cancer stage at time of diagnosis in comparable settings is similarly hard to establish.

In South-Africa, Lomalisa et al. found that HIV-positive patients with a CD4 count of below 200/mm3 had significantly more advanced tumor stages than HIV-negative women [9]. Fruchter et al. found HIV-positive patients to be at elevated risk for advanced cervical cancer diagnoses in univariate analysis. That said, their observed study group was small and statistical significance ceased after adjusting their model for other variables [8]. Moodley et al. found advanced stages among both HIV-positive and -negative patients with no association between HIV-status and stage [10]. On average HIV-seropositive women in our study presented 11 years younger than patients with a negative or unknown HIV-status [14]; this is consistent with the previous scientific literature where HIV-positive women presented 10–15 years earlier than HIV-negative women [10, 30]. The association of HIV-infection and advanced stage presentation of cervical cancer could potentially be explained by the HIV-associated immunodeficiency leading to a more rapid cancer growth, although other authors attribute this to molecular interactions between HIV and HPV [31–33]. Ibrahim et al. in Sudan identified high age and rural residence as predictors for advanced stage presentation [16]. Findings from similar studies in Morocco and South India include socioeconomic factors such as low education and illiteracy [17, 19].

In our study, women from rural areas tended to have longer patient intervals. This may be attributable to a low awareness of cervical cancer and its associated symptoms or the lack of health facilities and skilled personnel in rural parts of the country. Macleod et al. reported a lack of awareness and a misinterpretation of the seriousness of symptoms as the main risk factor for long patient intervals [20]. Fear of finding cancer and socioeconomic factors like illiteracy were other common themes among comparable studies [11, 17, 21].

In multivariate analysis, rural origin was associated but both age and HIV-status were not associated with long patient intervals in our study. Other factors that presumably influence the length of the patient interval include financial and logistic factors including delays in health care service.

We observed long time periods of median 25 to 35 weeks between women noticing the first symptom and pathological diagnosis among all stages, increasing with FIGO-stage. A comparable study in Nepal found shorter patient intervals of median 22 weeks [18]. Even in early stages, women were often diagnosed after many weeks or months of experiencing symptoms of cervical cancer such as abnormal vaginal bleeding, pain and vaginal discharge. After pathological diagnosis, patients often had to wait months until radiotherapy started, which further increased the risk of cancer progression [6].

The key strength of this study is the large sample size with patients coming from all over Ethiopia. There are, however, certain limitations we need to acknowledge. Data regarding patient and tumor characteristics and dates used for the calculation of patient interval were extracted from handwritten medical records. These dates relied on self-reporting from patients who might have been subjected to recall bias. We do not know how well women remembered the date of symptom onset and how meticulously it was documented. Secondly, women who were symptomatic for a long time but never presented to a health care professional may have died at advanced stages without ever being diagnosed at TASH and thus did not appear in our study. Hence, such a selection bias might falsely result in favorable data, particularly for patients with advanced cancer stages. Since the date of first presentation to a health care professional was not available for many patients, it was not possible to identify precisely at which stages of the diagnostic pathway these delays occur. Some patients may have been clinically diagnosed earlier and then had to wait until referral for pathological diagnosis – yet for the patients for whom all data were available, this interval did not vary substantially between tumor stages and residence. Qualitative research is needed to identify the impediments to diagnosis which lead to long patient intervals and more advanced stage presentation.

Previous studies found that efforts in down-staging helped to significantly increase overall survival. A three-year program in rural Tanzania with the aim of down-staging cancer through proactive visits from trained health aides into people’s homes showed favorable results [34]. One study conducted in rural India found that a cervical cancer education group effectively reduced the ratio of advanced stage diagnoses and increased the number of women diagnosed at early tumor stages [35]. However, awareness of cervical cancer and knowledge of risk factors, signs and symptoms are still low among women in Ethiopia and other African countries [36–38]. In Malaysia, advanced cervical cancer presentation (stages III and IV) dropped from 60 to 26% within 4 years after a program was introduced, focusing on training health staff and strengthening public awareness through the use of pamphlets and posters in clinics and hospitals [39].

Prevention and down-staging programs could be integrated in HIV/AIDS care programs and other preexisting healthcare infrastructures like it has been successfully implemented in Zambia [40, 41]. Alongside an increased coverage of HPV vaccination and screening, such initiatives could help reduce cervical cancer mortality and incidence worldwide.

## Conclusion

Our results support the hypothesis that long patient intervals lead to more advanced cervical cancer stages at pathologic diagnosis. Especially rural women tended to be diagnosed late and need to be addressed through awareness programs. HIV-postive women were at elevated risk of advanced tumor presentation; this should encourage efforts of the government to implement specific screening programs for HIV positive women. In addition to the current government efforts to implement nationwide screening programs, information about signs and symptoms of the disease should be spread.

## Data Availability

The datasets analyzed are available from the corresponding author on reasonable request.

## Abbreviations

CI: Confidence Intervals
FIGO: International Federation of Gynecology and Obstetrics
HIV: Human Immunodeficiency Virus
HPV: Human Papilloma Virus
HR: Hazard Ratio
TASH: Tikur Anbessa Specialized Hospital
